# Slant of a Surface Shifts Binocular Visual Direction

**DOI:** 10.3390/vision2020020

**Published:** 2018-05-06

**Authors:** Tsutomu Kusano, Koichi Shimono

**Affiliations:** 1 Faculty of Human Sciences, Kanagawa University, Yokohama-shi, Kanagawa Prefecture 221-8686, Japan; 2 Graduate School of Marine Science and Technology, Tokyo University of Marine Science and Technology, Koto-ku, Tokyo 135-8533, Japan

**Keywords:** visual direction, slanted surface, inclined surface, binocular disparity stimuli, depth cue conflict

## Abstract

We demonstrate how the slant of a surface affects the relative visual direction between binocular stimuli. In two experiments, we measured the visual direction of a binocular stimulus at different distances in the mid-sagittal plane or in the transverse plane at eye level relative to the center of the stimulus field. Experiment 1 showed that when a binocular stimulus (a vertical bar) was presented in front of or behind a surface slanted along the vertical center of the surface, its visual direction shifted toward the surface. Experiment 2 showed that when a binocular stimulus (a horizontal bar) was presented in front of or behind a surface slanted along the horizontal center of the surface, its visual direction also shifted toward the surface. These results indicate that the slant of a surface should be listed among the variables that contribute to the binocular visual direction, as well as the retinal loci of the stimulus, binocular eye position, the location of the visual egocenter, and stimulus properties.

## 1. Introduction

Historically, the visual direction of a binocular stimulus was known to be determined by three variables—retinal loci of the stimulus, binocular eye position, and the location of the visual egocenter (e.g., [1,2,3])—and there is ample evidence supporting this idea (see [4,5,6]). Differences in stimulus properties between the two eyes are also known to be variables that affect the visual direction of a binocular stimulus, such as blur [7], luminance [7,8,9,10], contrast [9,11,12], and binocular disparity [13]. In the present study, we show that the slant of a surface, either in front of or behind a binocular stimulus is a variable that affects binocular visual direction. We use the term “slant” as defined by Stevens [14] to describe the rotation of a surface (cf. [4]).

To show the effects of the slant of a surface, we examined the horizontal relative visual direction of a vertical bar in Experiment 1, and the vertical relative visual direction of a horizontal bar in Experiment 2. In Experiment 1, the vertical bar (standard stimulus) had horizontal disparity and was placed either in front of, or behind a surface rotated along its vertical center. In Experiment 2, the horizontal bar (standard stimulus) had horizontal disparity and was also placed either in front of, or behind a surface rotated along its horizontal center. Observers were asked to adjust the horizontal (Experiment 1) or vertical (Experiment 2) position of a zero-disparity stimulus (comparison stimulus), while maintaining their fixation so that it appeared aligned with the standard stimulus.

## 2. Materials and Methods

### 2.1. Apparatus

We used a MacBook Pro computer (Apple Inc., Cupertino, CA, USA) running MATLAB (MathWorks, Natick, MA, USA) with the Psychtoolbox extension [15,16,17] for stimulus generation, experiment control, and recording observers’ responses. The stimuli were stereograms consisting of two half-images presented side-by-side on a 17-inch Mitsubishi RDF173H CRT monitor (Mitsubishi Electric, Tokyo, Japan), which was gamma-corrected with a Minolta LS-100 luminance meter (Konica Minolta, Tokyo, Japan). A double-mirror stereoscope was used and the optical distance was 114 cm. The observer’s head was supported by a head-and-chin rest.

### 2.2. Stimuli

**Figure 1:**
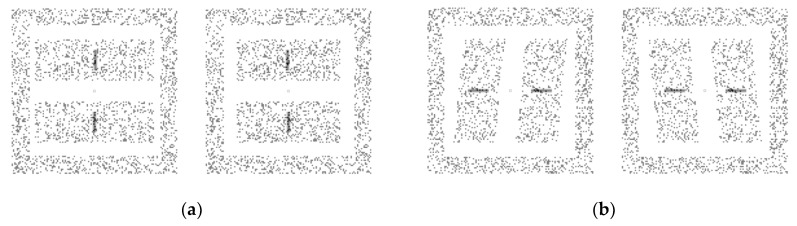
Schematics of the stereograms used in (**a**) Experiment 1 and (**b**) Experiment 2—The color is inverted (i.e., actual stimuli were white dots on a black background.) (**a**) Two rectangular areas depict two surfaces slanted about the vertical axis whose angles are identical. When the stereogram is viewed by crossing the eyes, the right side of the surface appears farther from the observer. (**b**) Two parallelogramatic areas depict two surfaces slanted about the horizontal axis whose angles are identical. When the stereogram is viewed by crossing the eyes, the top side of the surface appears farther from the observer.

Figure 1a schematically depicts the stereogram used in Experiment 1, which consisted of two rectangular areas (upper and lower) each containing a vertical bar, a square frame area surrounding the rectangular areas and the bars, and a central fixation point in each half-field. The rectangular areas and the frame area contained randomly placed white dots on a dark background. The luminance of each dot was 64.7 cd/m^2^ and that of the background was 1.38 cd/m^2^. The outer dimensions of the frame area, defined by the dots, was 7.81° (degrees of visual angle) in width and height, and that of its inner dimension was 6.25° in width and height. The dot density of the frame area was 51.2 dots/deg^2^. The fixation point was a small filled circle with a 3.8-arcmin diameter, and it was placed in the center of the frame area in each half-image. Thus, the fixation point was in the mid-sagittal plane and in the transverse plane at eye level. The fixation point also had zero disparity with respect to the monitor. The purpose of the fixation point and the frame area was to aid observers in maintaining their convergence.

Each of the two rectangular areas of the stereogram had the same horizontal size disparity. To create a disparity, we first distributed dots randomly within the “original” rectangular areas, and then re-assigned the horizontal positions of the dots in one half-image without changing their vertical positions or size. The size of each original “rectangular” area was 5.0° in width and 2.0° in height. The upper and lower original rectangular areas were vertically separated from each other by 1.0°. The dot density was 51.2 dots/deg^2^. To calculate the horizontal position of each dot, we used the van Ee and Erkelens’s equation [18]. The manipulation of the horizontal dot position created a 10.2% size enlargement of the rectangular areas in one-half image, while the other half-image remained constant in size. Thus, the dot density in the rectangular areas in one-half image became 46.5 dots/deg^2^, while that in the other half-image was constant (51.2 dots/deg^2^). The horizontal size disparity corresponded to a slant of 60° along the vertical axis of the rectangular areas with respect to the fronto-parallel plane. When the areas were fused, two surfaces, which slanted about the vertical axis with the same angle, would be perceived. The center of the slant (the vertical center of the rectangular area) had zero binocular disparity with respect to the monitor or fixation plane.

In Experiment 1, the lower and upper fused bars were used as the standard and comparison stimuli (bars), respectively. We did not counterbalance the positions of the standard and comparison bars to shorten the experiment duration; our preliminary studies showed that the counterbalancing did not affect the obtained results. The standard bar had one of five distances (−9.6, −4.6, 0.0, 4.3, or 8.2 cm), which corresponded to a disparity of −15.0, −7.5, 0.0, 7.5, or 15.0 arcmin, respectively. Negative and positive distance values represent the standard bar behind or in front of the fronto-parallel plane (uncrossed and crossed disparities) relative to the fixation point, respectively. To present the standard bar in front of or behind the fronto-parallel plane, we shifted its horizontal position in each half-image outward or inward with respect to the fixation point. The absolute amount of shift in each half-image was identical, but the direction of shift was opposite for the positive and negative disparities; the standard bar was always presented on the mid-sagittal plane. The comparison bar had zero horizontal disparity relative to the fixation point, and its horizontal position was moveable. The initial horizontal position of the comparison bar was randomly set to a value in the ranges of 8.5-arcmin to the left to 8.5-arcmin to the right with respect to the vertical center of the rectangular areas in 0.95-arcmin steps. The horizontal position of the comparison bar was adjusted by 0.2-arcmin steps. When the standard bar had zero disparity, a center-to-center separation between the standard and comparison bars was 3.0 degrees. We assumed that Panum’s fusional range for our stimulus (bar) was larger than that traditionally reported for a line segment (e.g., [19]), because the bars used in the current study were embedded in a random dots pattern. For studies measuring the fusional range for the random dots patterns, see e.g., [20,21].

Figure 1b schematically depicts the stereogram used in Experiment 2 that consisted of two parallelogrammatic areas (right and left), each containing a horizontal bar, a square frame area surrounding the parallelogrammatic areas and the bars, and a central fixation point in each half-field. The parallelogrammatic areas and the frame area contained randomly placed white dots on a dark background. The luminance of each dot and that of the background were each the same as those of the stereogram used in Experiment 1. The purpose of the fixation point, the framed area, and their stimulus properties were all identical in Experiment 1.

Each of the two parallelogrammatic areas had the same horizontal-shear disparity. To create the disparity, we first distributed dots randomly within the “original” rectangular areas, and then re-assigned the horizontal positions of the dots in one half-image without changing their vertical positions and size. The size of each original rectangular area was 5.0° in width and 2.0° in height. The upper and lower original rectangular areas were vertically separated from each other by 1.0 deg. The dot density was 51.2 dots/deg^2^. To calculate the horizontal position of each dot, we used the van Ee and Erkelens’s equation [18] as in Experiment 1. The manipulation of the horizontal dot positions created parallelogrammatic areas so that their shear angles were 5.6° clockwise in one half-image and 5.6° counterclockwise in the other half-image. The horizontal-shear disparity corresponded to a slant of 60.0° along the horizontal axis of the parallelogrammatic areas with respect to the fronto-parallel plane. When the areas were fused, two surfaces that slanted about the horizontal axis with the same angle would be perceived. The center of the slant (the horizontal center of the parallelogrammatic areas) had zero binocular disparity with respect to the monitor or fixation plane.

In Experiment 2, the left and right fused bars were used as the standard and the comparison bars, respectively. As in Experiment 1, we did not counterbalance the positions of the standard and comparison bars. The standard bar had one of seven distances (−15.0, −9.6, −4.6, 0.0, 4.3, 8.2, or 11.9 cm), which corresponded to a disparity of −22.5, −15.0, −7.5, 0.0, 7.5, 15.0, or 22.5 arcmin, respectively. As in Experiment 1, negative and positive distance values represent the standard bar either behind or in front of the fronto-parallel plane, uncrossed and crossed disparities, relative to the fixation point, respectively. To present the standard bar in front of or behind the surface, we shifted its horizontal position in each half-image outward or inward with respect to the point 3.0 degree left from the fixation point. The absolute amount of shift in each half-image was identical, but the direction of shift was opposite for the positive and negative disparities; the standard bar was always presented on the horizontal plane of the eyes. The comparison bar was moveable and had zero horizontal disparity relative to the fixation point as in Experiment 1. The initial vertical position of the comparison bar was randomly set to a value in the range of 8.5 arcmin upward to 8.5 arcmin downward with respect to the horizontal center of the rectangular areas in 0.95-arcmin steps. The vertical position of the comparison bar was adjusted by 0.2-arcmin steps. When the standard bar had zero disparity, a center-to-center separation between the standard and comparison bar was 3.0°. As in Experiment 1, we assumed that Panum’s fusional range for our stimulus (bar) was larger than what was traditionally reported (e.g., [19]), although the range of disparity used in Experiment 2 was slightly larger than used in Experiment 1.

In each of the two experiments, each bar embedded in the rectangular or parallelogrammatic areas was constructed by windowing a 12 cycles per deg (cpd) cosine grating with a rectangular window of 1/12-deg width. The length of the bars was 1.0° in both Experiments 1 and 2. The luminance of a pixel located at *x* (horizontal position in Experiment 1 and vertical position in Experiment 2 relative to the bar’s center) in the bars *L*(*x*) was calculated as follows:

(1) L(x)=0.5[1+a∗cos(2πfx), if abs(x)<2.5, else 0

where *a* is the amplitude and *f* is the spatial frequency. In this experiment, *a* was 0.96 and *f* was 12 cpd. The bars were anti-aliased in every frame of the monitor. The maximum luminance of the bar was 64.7 cd/m^2^ and the minimum was 1.38 cd/m^2^: the same luminance as the monitor background. The luminance of the random dots was added linearly to that of the bars if they overlapped, so that the resultant luminance could exceed the maximum luminance described above. When the stereogram was fused, (1) the bars in front of the surface appeared translucent so that the fused dots were seen at the surface though the bars, and (2) the bars behind the surface were seen through the fused dots, which appeared translucent at the surface.

### 2.3. Procedure

In each experiment, four alignment estimates were collected for each combination of slant conditions and distances in depth (horizontal disparities) of the standard bar. This resulted in 60 trials (4 estimates × 3 surface slants × 5 distances) in Experiment 1 and 84 trials (4 estimates × 3 surface slants × 7 distances) in Experiment 2. The presentation order of the trials was randomized for each observer. The positions of the dots in the rectangular and parallelogrammatic areas as well as the frame area were distributed and rearranged randomly across trials. Before each experiment, each observer performed several practice trials, which were randomly selected from trials in the main experiment, until the experimenter judged that the observer understood the task.

Before the start of each trial, we presented the fixation point. The observer’s first key press presented the rectangular and parallelogrammatic areas as well the square frame area, and their second key press presented the standard and comparison bars. The method of adjustment was used to measure the visual direction of the standard bar. Observers were instructed to adjust the horizontal (Experiment 1) or vertical (Experiment 2) position of the comparison bar with key presses until it appeared to be aligned with the standard bar or to be seen in the same visual direction. When the adjustment was completed, the observer pressed the space bar to terminate the trial, which immediately extinguished the stimuli except for the fixation point. The presentation time of the standard and the comparison bars was unlimited, but usually lasted approximately 15 s, at most, for each trial. We referred to the adjusted position of the comparison bar as the (relative) visual direction that was expressed in the angular difference between the adjusted position and the mid-sagittal plane in Experiment 1, or between the adjusted position and the transverse plane at eye level in Experiment 2.

### 2.4. Observers

Five and six observers participated in Experiments 1 and 2, respectively. Two observers (one of them being the first author) participated in both experiments. All reported that they had normal or corrected-to-normal visual acuity (more than 20/20), and Titmus test showed that they had stereo-acuity of less than 100 arcsec. Everyone but the first author was naive as to the purpose of the experiment. All observers provided their informed consent for voluntary participation in the experiments. The experiments were carried out in accordance with the Code of Ethics of the World Medical Association (Declaration of Helsinki).

## 3. Results

### 3.1. Experiment 1 Results

We coded each adjusted position of the comparison bar in terms of its angular distance from the horizontal center of the stereogram (see Figure 1a), and the mean of four measurements for each of the five observers in each subcondition was the basic unit of analysis. We used a two-way repeated measures analysis of variance (ANOVA) to analyze the effects of the surface slant, distance in depth (or horizontal disparity) between the standard and comparison bars, and their interaction on the mean. There were three slant angle conditions: right-far, right-near, and fronto-parallel. In the right-near condition, the right side of the surface appeared closer to the observer; in the right-far condition, the right side of the surface appeared farther from the observer; in the fronto-parallel condition, the surface appeared on the fixation plane. There were five distance conditions: the standard bar appeared in front of the comparison bar in two conditions, behind the comparison bar in another two conditions and at the same distance as the comparison bar in the last condition.

**Figure 2:**
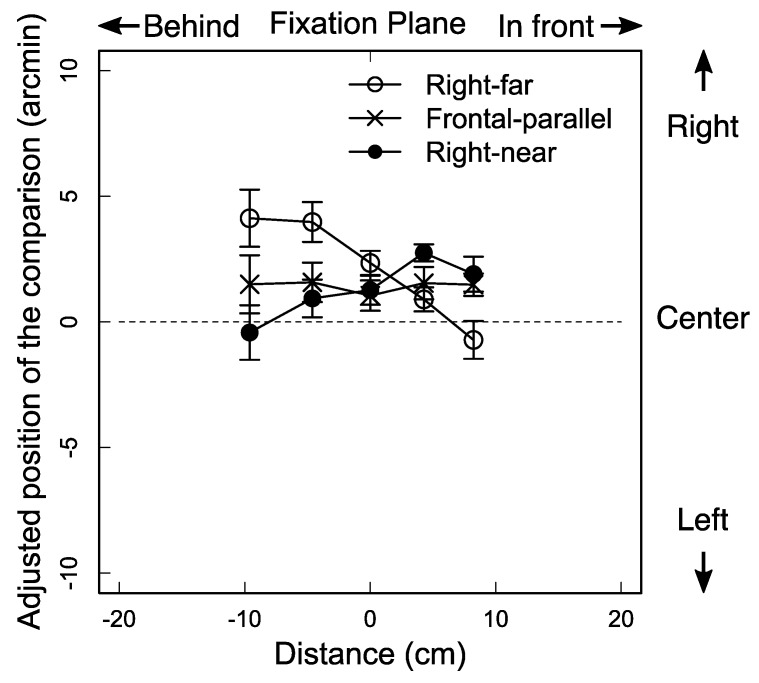
Results from Experiment 1. Mean horizontal positions, over five observers, of the comparison bar as a function of distance of the standard bar in depth from the fixation plane for the three slant conditions. Positive and negative numbers in ordinate indicate rightward and leftward deviations of the adjusted position of the comparison bar, respectively, from the vertical center of the rectangular areas depicting the surface. Positive and negative numbers on abscissa indicate the distance of the standard bar in front of and behind the fixation plane, respectively. Error bars denote the standard error of the mean (SEM).

The ANOVA (3 slants × 5 distances) showed that their interaction was statistically significant [*F* (8, 32) = 22.38, *p* < 0.001, general *η*^2^ = 0.06] and that the main effect of the slant was statistically significant [*F* (2, 8) = 18.19, *p* < 0.005, general *η*^2^ = 0.04], while the main effect of distance was not. The significant interaction is depicted in Figure 2: the mean over the observers decreased as a function of distance of the standard bar for the right-far condition, whereas it increased for the right-near condition in general. The significant main effect can be seen in the differences among the means across the five distances for the three slant conditions; the mean was 2.13 arcmin [Standard Deviation (*SD*) = 2.01] in the right-far condition, that was 1.43 arcmin (*SD* = 0.22) in the fronto-parallel condition, and that was 1.29 arcmin (*SD* = 1.18) in the right-near condition. Figure 2 also shows that most of the data points appear above the vertical center. Using Abdi’s method [22], we performed a two-way repeated measures ANOVA (3 slants × 5 distances) with effect coding. The results showed that the grand mean was significantly different from zero [*F* (1, 32) = 344.84, *p* < 0.001]. We do not have a good explanation for this rightward bias.

The results show that the horizontal direction of a binocular stimulus depends on the sign of surface slant about the vertical axis. Specifically, the results indicate how the sign of the slant affects the binocular visual direction: (1) a binocular stimulus in front of the surface shifts more leftward (clockwise from the top view) in the right-far condition than in the fronto-parallel condition, and it shifts more rightward (counterclockwise) in the right-near condition than in the fronto-parallel condition; (2) the stimulus behind the surface shifts more rightward (clockwise) in the right-far condition than in the fronto-parallel condition, and it shifts more leftward (counterclockwise) in the right-near conditions than in the fronto-parallel condition; finally, (3) the stimulus on the surface shifts almost the same among the three slant conditions (see Figure 2). These results indicate that the visual direction of a binocular stimulus, whether in front of or behind a surface, shifts toward the slanted surface.

**Figure 3:**
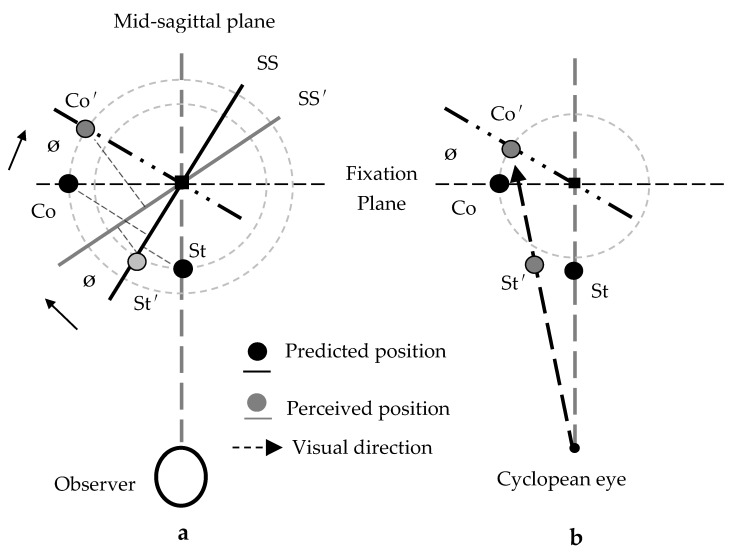
Schematics of perceived surface (SS′), perceived standard bar (St′), and perceived comparison bar (Co′) from the top view of the right-far condition in Experiment 1. In (**a**), the amount of slant of SS′ is underestimated compared to that of a surface (SS) predicted by the horizontal size disparity cue. The angle θ is the amount of underestimation. St′ appears to shift leftward from the mid-sagittal plane, where the standard bar (St) is supposed to be positioned, as if St were attached to the perpendicular line toward SS. Similarly, Co′ appears to shift backward from the fronto-parallel plane, where the comparison bar (Co) is geometrically predicted to be positioned, when Co is left of the mid-sagittal plane. In (**b**), the observer adjusts Co, so that St′ and Co′ are aligned. During adjustment, Co′ appears to move along a frontal plane rotating around the fixation point, so that its left side is far from the fronto-parallel plane and its rotating angle becomes θ. As seen in the figure, as long as the angle θ is not too large, the adjusted position of Co corresponds well with the visual direction of St′. We created this figure under the assumption that the visual direction is judged from a cyclopean eye located at the midpoint between the eyes (e.g., [27,28,29]).

The results are explained by the amount of the perceived slant of a surface and the relative horizontal disparity between the standard bar and the surface. The stereogram used in this experiment contains conflicting depth cues in the central areas; the horizontal size disparity cue indicates a slanted surface along the vertical axis away from the fronto-parallel plane, while other depth cues such as perspective and the texture gradient indicate a surface in the fronto-parallel plane. If the horizontal size disparity cue becomes less reliable when it is presented with other conflicting depth cues in a surface stimulus, the amount of the perceived slant of the surface is likely to be less than that simulated by the size disparity (e.g., [23,24]). Furthermore, if the visual system treats the horizontal relative disparity (i.e., depth) between the surface and a small binocular object in its vicinity as reliable (e.g., [25,26]), the object would be positioned as if it “shifts” with the surface whose amount of slant is underestimated. These arguments are consistent with the shift of the relative visual direction observed in this experiment. If a binocular stimulus in front of or behind a slanted surface shifts together with the surface to maintain a constant relative depth between them, the visual direction of the standard bar would depend on the sign of the surface slant; for example, when a surface slants anti-clockwise from the fronto-parallel plane from the top view, the standard bar in front of the surface would shift leftward (see Figure 3a) and the standard bar behind it, rightward. It is as if the standard bar were attached to the perpendicular line toward the surface and rotated around the fixation (see Figure 3a).

If the underestimation of a surface slope affects the position of the standard bar, it will also affect the perceived position of the comparison bar. Our geometrical analysis (see Figure 3) indicates that (1) the perceived position of the comparison bar is away from the fronto-parallel plane, where the bar is supposed to be positioned, and (2) it would be on a plane that slants at the fixation point, making the angle from the fronto-parallel plane the same as that of the underestimation. This suggests that the adjusted position of the comparison bar corresponds rather well with the visual direction of the standard bar as long as the angle of the underestimation is not so large. This suggestion is consistent with the fact that when a slanted surface is surrounded by the fronto-parallel plane, as in the stereograms we used (see Figure 1), the underestimation is not so large [18].

### 3.2. Experiment 2 Results

We coded each adjusted position of the comparison bar in terms of the angular distance from the vertical center of the stereogram and the mean of four measurements; this was done for each of the six observers, with each subcondition being the basic unit of analysis, as in Experiment 1. A two-way repeated measures ANOVA was used to analyze the effects of the surface slant, the distance in depth (or horizontal disparity) between the standard and comparison bars, and their interaction on the mean. There were three slant conditions: top-near, top-far, and fronto-parallel. In the top-near condition, the upper side of the surface appeared closer to the observer; in the top-far condition, the upper side of the surface appeared farther from the observer; in the fronto-parallel condition, the surface appeared on the fixation plane. There were seven distance conditions; the standard bar appeared in front of the comparison bar in three conditions, behind the comparison bar in another three conditions, and at the same distance as the comparison bar in the last condition. We increased the distance intervals between the standard and the fixation planes in Experiment 2 compared with those in Experiment 1 to better examine its effects on the visual direction.

**Figure 4:**
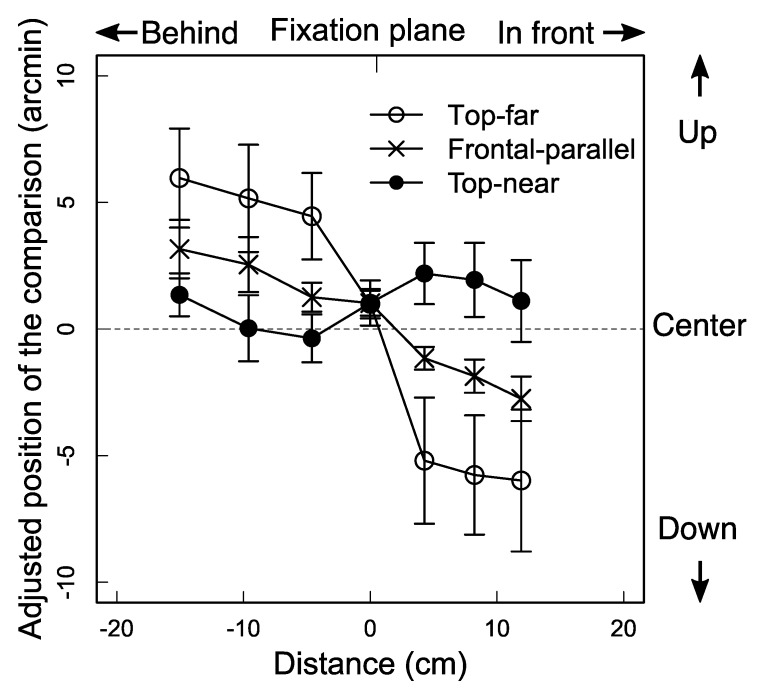
Results from Experiment 2. The mean vertical positions, over six observers, of the comparison bar as a function of distance of the standard bar in depth from the fixation plane for the three slant conditions. Positive and negative numbers in ordinate indicate upward and downward deviations of the adjusted position of the comparison bar, respectively, from the horizontal center of the parallelogrammatic areas depicting the surface. Positive and negative numbers on abscissa indicate the distance of the standard bar in front of and behind the fixation plane, respectively. Error bars denote the SEM.

The ANOVA (3 slants × 7 distances) showed that the interaction was statistically significant [*F* (12, 60) = 5.99, *p* < 0.001, general *η*^2^ = 0.35] and the main effect of distances was also statistically significant [*F* (6, 30) = 10.98, *p* < 0.001, general *η*^2^ = 0.37], while the main effect of the slant was not. The significant interaction is depicted in Figure 4; the mean over the six observers decreased as a function of the distance of the standard bar in the top-far condition, while, in the top-near condition, it was relatively constant. The significant main effect can be seen in Figure 4, where the mean decreases as a function of the distances of the standard bar as a whole.

The results show that the vertical visual direction of a binocular stimulus depends on the sign of the surface slant about the horizontal axis. Specifically, the results indicate how the slant sign affects the binocular visual direction: (1) a binocular stimulus in front of the surface shifts more downward (counterclockwise from the right-side view) in the top-far condition than in the fronto-parallel condition and shifts more upward (clockwise) in the top-near condition than in the fronto-parallel condition; (2) the stimulus behind the surface shifts more upward (counterclockwise) in the top-far condition than in the fronto-parallel condition and shifts more downward (clockwise) in the top-near condition than in the fronto-parallel condition; and (3) the stimulus on the surface shifts almost the same among the three slant conditions. These results indicate that the visual direction of a binocular stimulus in front of or behind a surface slanted about the horizontal axis shifts toward the surface.

The results are explained by the amount of perceived slant of a surface and the relative horizontal disparity between the standard bar and the surface, as in the results of Experiment 1. This differs from Experiment 1, however, in that the amount of perceived slant is determined by two factors: One is the depth cue conflict described earlier (e.g., [23,24]), and the other is perceived geographical slant (e.g., [30,31,32,33]). As in Experiment 1, the stereogram in this experiment contained conflicting depth cues in its central areas; the horizontal-shear disparity cue indicates a surface slanted along its horizontal axis away from the fronto-parallel plane, while other depth cues, such as perspective and the texture gradient indicate a surface in the “fronto-parallel” plane. If the conflicting cue makes the effectiveness of the horizontal shear-disparity less reliable, the amount of the perceived slant of the surface is likely to be less than that simulated by the disparity (e.g., [18,23]). Moreover, it has been proposed that when the visual information on a ground surface is insufficient, as in this experiment, the visual system assumes the implicit ground surface slants uphill (e.g., [32]). If the apparent fronto-parallel plane is perpendicular to the implicit ground surface, it rotates from the physical fronto-parallel plane toward the top-near plane. We assume here that depth cue conflict and geographical slant operate in the same direction or in opposite direction, and the two factors operate either additively or subtractively.

**Figure 5:**
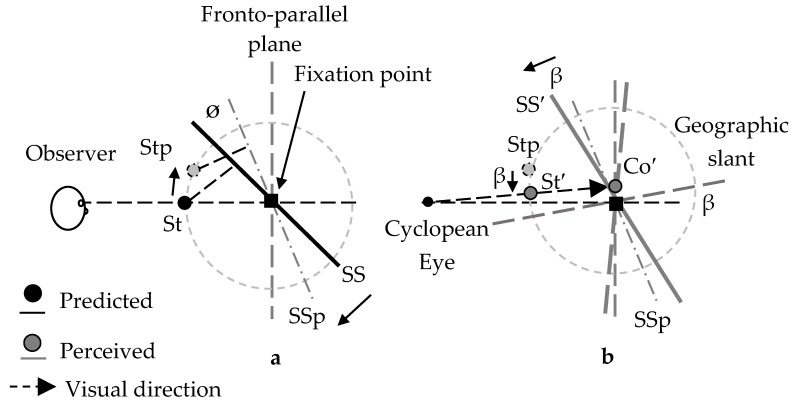
Schematics of perceived surface (SS′), perceived standard bar (St′), and perceived comparison bar (Co′) from the right view of the top-near condition in Experiment 2. In (**a**), SSp and Stp indicate the “positions” of a surface and a standard bar, respectively, which are expected from the underestimation of surface slant (SS). The angle θ is the amount of underestimation. In (**b**), the geographical slant is assumed to rotate around the fixation point so that it slants uphill. The angle β is the amount of geographical slant. St′ appears to shift downward from Stp, as if it shifts with geographical slant. When the observers adjust the position of the comparison bar, Co′ appears to move along a plane rotating around the fixation, as if the angle θ is subtracted from the angle β; for example, when θ is larger than β, the plane rotates from the fronto-parallel plane toward the top-far plane. As seen in the figure, as long as the angle θ is not so large, the position of the adjusted comparison bar corresponds rather well with the visual direction of St, as long as the angle of the underestimation is not too large.

Let us assume that when a small binocular object is presented with a slanted surface, the object appears to “shift” its position with the surface, so as to keep the horizontal disparity between them constant as assumed in Experiment 1 [25,26]. In the top-far condition, if the two factors (depth-cue conflict and graphical slant) operate in the same direction, the apparent slant would shift toward the fronto-parallel plane. In the top-near condition, if they operate in the opposite direction, the apparent slant shift would be smaller than that in the top-far condition (see Figure 5): For example, if the extent of the apparent shift induced by conflicting depth cues and that induced by the geographical surface are the same, there would be no effect. In the fronto-parallel condition, the depth cue conflict has no effect and the geographical surface operates, and then, the apparent slant would shift in the same direction as the geographical slant. If a binocular stimulus in front of or behind a slanted surface shifts together with the surface, the results of Experiment 2 can be explained.

The idea of how the two factors operated in Experiment 2 is consistent with the fact that the amount of the visual direction shift observed in Experiment 2 was larger than that observed in Experiment 1 (compare Figure 2 and Figure 4). As can be seen in Figure 2 and Figure 4, the amount of the shift was around 5 arcmin in Experiment 1 and was around 12 arcmin in Experiment 2. The difference in the shift can be explained by the assumption that when the two factors (depth-cue conflict and graphical slant) operate in the same direction, the amount of the visual direction shift is increased, as in the top-far condition in Experiment 2, while in Experiment 1, only one factor (depth cue conflict) was operational.

We discuss here whether or not the difference of disparity used between Experiments 1 and 2 played a role in the different results from the two experiments within the framework of our reasoning. The discussion is interesting, because some perceptual properties are often reported to be different between horizontal-size disparity, which was used in Experiment 1, and horizontal-shear disparity, which was used in Experiment 2 [34,35]. According to our reasoning discussed above, the different results can be explained by the difference in disparity used, if the amount of the perceived slant, which is underestimated from what was predicted by disparity, is larger in Experiment 2 than in Experiment 1. The literature tells us that while the slant of a surface along the horizontal axis is known to be “typically more severely underestimated than” that along the vertical axis (See Figure 20.32 in p.413 of [4]), underestimation is also known to be less prominent when a slanted surface is presented with the fronto-parallel plane as in the stereograms we used (see Figure 1) as discussed previously [36]. These arguments suggest that the difference of disparity used may not have been an important factor leading to the different results of the two experiments.

One might think that the factor operating in this experiment was not a geographical slant, but rather the Visually Perceived Eye Level (VPEL), which is assumed to be a reference to the elevation of an object. VPEL is known to shift from the observer’s true eye level as a function of the slant of a surface that rotates along the horizontal axis: VPEL shifts upward, when the upper side of the surface slants away from the observer, as in the top-far condition, and it shifts downward when it appears closer to the observer, as in the top-near condition [37,38,39]. Accordingly, it is often assumed that when VPEL shifts upward, the apparent height of a stimulus on a surface-near gaze is underestimated in the top-far condition and overestimated in the top-near condition [37,39]. This assumption predicts that “if VPEL is mistaken for true horizontal” (O′Shea and Ross [39], p. 1170), the standard bars appear downward from a plane in the top-far condition and upward from a plane in the top-near condition. As seen in Figure 4, the data are not consistent with the prediction. Thus, the present result is difficult to explain using the factor of VPEL.

We think that cyclovergence (e.g., [4]) did not occur in the present experiment for two reasons. First, Rogers and Bradshaw [40] used scleral contact lenses and found that horizontal shear disparity induced little or no cyclovergence. We also conducted an additional experiment with a condition where the cyclovergence is supposed to be absent, and found similar results as those in Experiment 2. The stimulus used in the additional experiment had four slanted surfaces, placed side by side (two on either side of the fixation point), where the slant direction of the first and third surfaces (from the left) were opposite to the second and fourth. Judging from Rogers and Bradshow’s findings as well as our own, we have concluded that the present results cannot be explained in terms of cyclovergence.

## 4. Discussion

We showed in two experiments that the visual direction of a binocular stimulus is affected by the slant of a surface presented either in front of, or behind the stimulus. Experiment 1 showed that the horizontal visual direction of a vertical bar varied with the sign of a surface rotated along its vertical axis. Experiment 2 showed that the vertical visual direction of a horizontal bar varied with the sign of a surface rotated along its horizontal axis. The results are explained by assuming that both the binocular stimulus and the surface rotate together towards the fronto-parallel plane. An additional factor or geographical slant is needed to account for the result of Experiment 2.

The role of a surface on visual direction has also been reported for a monocular stimulus. Recent studies have shown that when a monocular image is embedded in a binocular random-dots pattern, its visual direction is affected by the binocular visual direction of the pattern (e.g., [41,42,43,44,45,46,47,48,49,50]). Furthermore, Ono, Mapp, and Howard [51] argued that a background presented with a given monocular stimulus can affect the visual direction of the stimulus. These studies, along with the present one, indicate that when a stimulus (monocular or binocular) is presented with a background or foreground, the visual direction of the stimulus is influenced by the properties of the background or foreground.

A close look at the literature reveals that slant surfaces have already been suggested to affect horizontal “absolute” visual direction of an object viewed in a pitch room (e.g., [49]) or in a natural environment (e.g., [50]). For example, Matin and Fox [49] reported that a stationary stimulus projected on the wall of the pitch room appears to move upwards or downwards if the wall is pitched top-far or top-near. They found that the “apparent motion” of the stimulus correlated with VPEL, which was assumed to be the reference to the elevation of an object, and they argued that the change of VPEL corresponded to the amount of the slope of the pitch room; for the top-far slant, the stimulus appeared to be above the VPEL, and for the top-near slant, the stimulus appeared to be below the VPEL. O′Shea and Ross [50] found that the perceived height of the downhill or uphill correlated with VPEL and argued that VPEL assimilates to the slant of the downhill or uphill. These findings suggest that a surface slant (or VPEL) has an effect on absolute visual direction.

As discussed in Section 2.1 and Section 2.2, the role of a slanted surface has been reported in the stereoscopic depth perception literature as well [25,26]. For example, when two side-by-side vertical binocular stimuli with zero disparity are presented on a surface slanted along the vertical axis, they do not appear in the same depth plane, but nearly parallel to the slanted surface, suggesting that the visual system utilizes relative disparity between the surface and the stimulus near the surface to locate it. Thus, the perceived position of a slanted surface affects the relative depths of the bars [25]. The previous studies, along with this present one, show that surface slant is a factor that affects the visual direction as well as depth perception of a binocular stimulus, which is presented near the surface.

Finally, the data from the current study have implications for the traditional views as to what variables determine the visual direction of a binocular stimulus. In the literature, retinal position, binocular eye position, and the location of the cyclopean eye have been regarded as such variables (see, [4,5,6] for review). Recently, properties of the stimulus (e.g., luminance, contrast, and disparity) have also been reported as variables influencing the perceived direction (see Introduction). The current data clearly show that there is another variable affecting the binocular visual direction, in addition to those reported. Therefore, the slant of a surface should be added to the list of variables determining binocular visual direction.
